# Understanding 3D vision as a policy network

**DOI:** 10.1098/rstb.2021.0448

**Published:** 2023-01-30

**Authors:** Andrew Glennerster

**Affiliations:** School of Psychology and Clinical Language Sciences, University of Reading, RG6 6AL Reading, UK

**Keywords:** 3D vision, coordinate transformations, hierarchical spatial representation, navigation

## Abstract

It is often assumed that the brain builds 3D coordinate frames, in retinal coordinates (with binocular disparity giving the third dimension), head-centred, body-centred and world-centred coordinates. This paper questions that assumption and begins to sketch an alternative based on, essentially, a set of reflexes. A ‘policy network’ is a term used in reinforcement learning to describe the set of actions that are generated by an agent depending on its current state. This is an untypical starting point for describing 3D vision, but a policy network can serve as a useful representation both for the 3D layout of a scene and the location of the observer within it. It avoids 3D reconstruction of the type used in computer vision but is similar to recent representations for navigation generated through reinforcement learning. A policy network for saccades (pure rotations of the camera/eye) is a logical starting point for understanding (i) an ego-centric representation of space (e.g. Marr’s (Marr 1982 *Vision: a computational investigation into the human representation and processing of visual information*) 2$\frac{1}{2}$-D sketch) and (ii) a hierarchical, compositional representation for navigation. The potential neural implementation of policy networks is straightforward; a network with a large range of sensory and task-related inputs such as the cerebellum would be capable of implementing this input/output function. This is not the case for 3D coordinate transformations in the brain: no neurally implementable proposals have yet been put forward that could carry out a transformation of a visual scene from retinal to world-based coordinates. Hence, if the representation underlying 3D vision can be described as a policy network (in which the actions are either saccades or head translations), this would be a significant step towards a neurally plausible model of 3D vision.

This article is part of the theme issue ‘New approaches to 3D vision’.

## 1. Introduction

Open your eyes and look around. It is natural to assume that your perception must depend on a 3D representation of the scene and that this is what allows you to interact with objects and move around. However, there is a growing body of evidence from machine learning demonstrating that 3D representations are not necessary for navigation, scene rendering from novel viewpoints or other tasks that have, hitherto, been assumed to require a reconstruction of the scene. In the biological literature, there is a long history of proposals suggesting that the brain uses non-3D, non-map-like representations that still allow us to operate in a 3D world [1–8].

Rather than reconstruction, the key claim in this paper is that 3D vision is best understood as being made up of something like a series of reflexes, i.e. a set of outputs (often actions) that are triggered in different situations, where these situations consist of a sensory component and a task or goal. In deep reinforcement learning, this set of contingencies is called a ‘policy network’. If an agent does not have a 3D representation of the scene then it must nevertheless compare the current state to some kind of stored representation and, as a result of comparing the two, it must generate an output. This seems very far from the idea of a 3D reconstruction of a scene. The purpose of this paper is to provide an outline of how a policy network could support the same behaviour as a system that uses a 3D reconstruction of the scene.

### (a) The implementation of policy networks in machine learning and the cerebellum

For reinforcement learning in machines, the goal of the agent is to learn a policy, *π*, that maximizes the expected value or return. At time *t*, the agent takes an action, **a_t_**, given its current state, **x_t_**. This action in the world leads to new input and a new state, **x**_**t+1**_. The optimal policy relating these is labelled, π*, and the corresponding maximum reward function V* [9]. Three-dimensional behaviours learned in this way include flying a quadcopter, navigating in a city or finding novel routes in a maze [10–12]. Usually, the subscript *t* is dropped and a ‘policy’ refers to the whole set of actions, **a**, in the context of a whole set of states, **x**, i.e. *π*(**a**|**x**). In this paper, I have replaced the state vector, **x**, with two separate elements: **s** which derives from the stimulus (retinal or otherwise) and **g** which carries a signal about the current task or goal.

**Figure 1.**
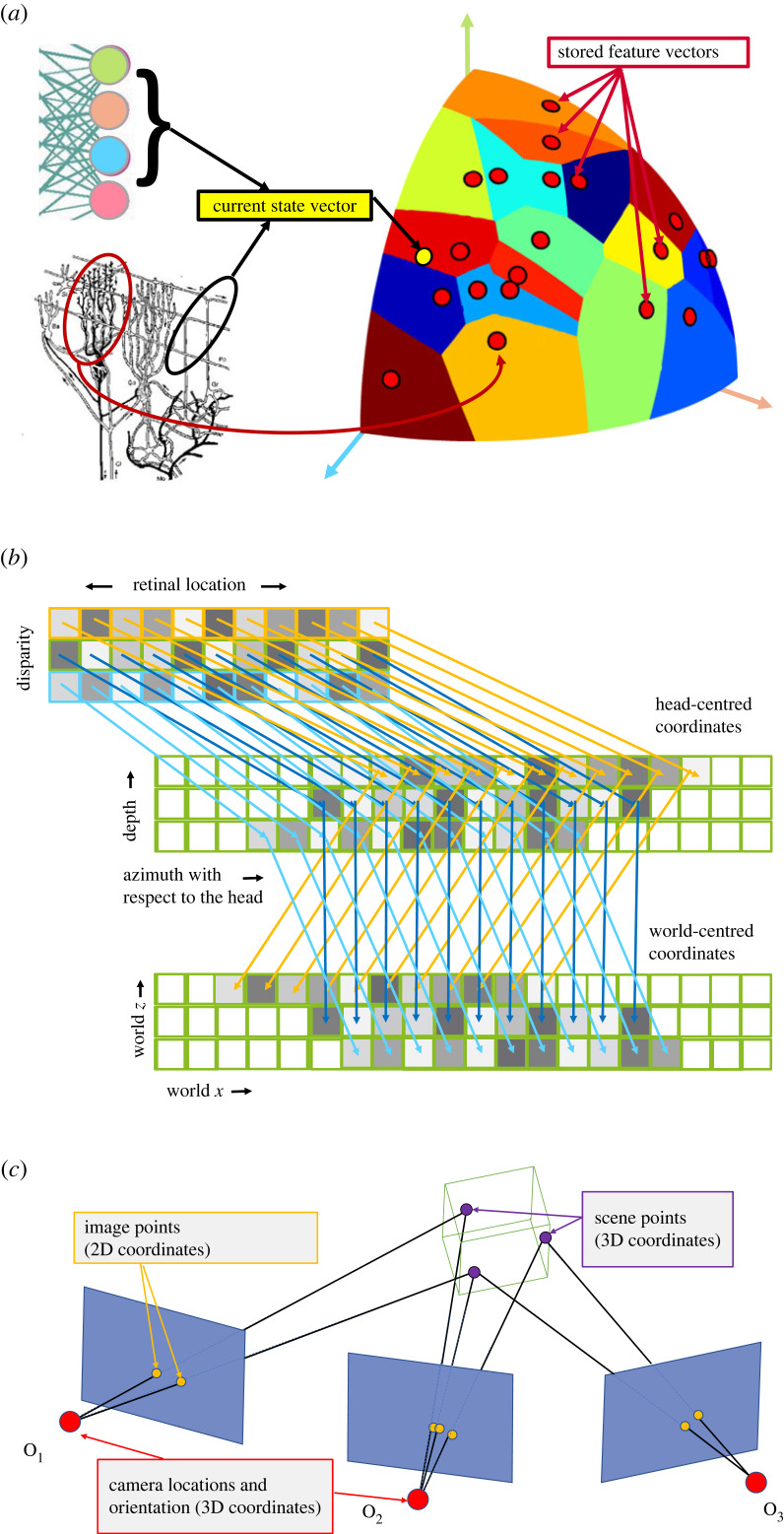
Policy networks and coordinate transformations in biology and machine vision. This figure illustrates the main hypotheses discussed in the paper. (*a*) The penultimate layer of a neural network provides a feature vector (current state vector) which is compared to a number of stored vectors (e.g. using Euclidean distance [15]) to determine the output. For ease of illustration, current and stored vectors are shown as unit vectors, so this comparison rule defines a set of Voronoi cells on a unit sphere with the stored feature vectors (red circles) as seeds. If a neural network has 4096 units in the penultimate layer (four shown above), then the current vector and stored features are $R^{4096}$. Purkinje cells in the cerebellum can be described in a similar way: they act as stored vectors (synaptic weights) that are compared to a current vector of incoming firing rates in parallel fibres. Current and stored vectors are in the same high-dimensional space and an action/output is chosen on the basis of similarity between them. For Purkinje cells, the dimensionality of the policy network (sphere) is approximately $R^{200000}$. (*b*) See text for a discussion of wholesale coordinate transformations of visual information. The top row indicates firing rates of neurons in a retinotopic area (e.g. primary visual cortex), where each square shows one output neuron and the grey level indicates its firing rate. Neurons with different retinal locations and different disparity tuning are shown. These firing rates would need to be transferred to neurons in other areas (e.g. posterior partietal cortex for a head-centred coordinate representation and hippocampus or entorhinal cortex for a world-centred coordinate frame). The mapping cannot be not fixed: it must depend on eye position with respect to the head and head orientation with respect to the world. (*c*) In computer vision, the goal of photogrammetry is to take a set of images captured by a moving camera and compute both the 3D structure of the scene and the path of the camera within it. For the computation involved in photogrammetry, there is no intermediate ‘body-centred’ coordinate frame corresponding to the putative posterior parietal cortex representations. (Online version in colour.)

In biology, similar ideas about the implementation of reinforcement learning have been described for many decades (e.g. [13,14]) although not using the language of policy networks. The cerebellum stores sensori-motor contingencies such that a certain sensory input will lead to a particular action and does so on the basis of the similarity between the synaptic weight vector of each Purkinje cell with the incoming firing rate vector. Figure 1*a* illustrates the close parallels between a policy network implemented by the cerebellum (according to Marr [13] and Albus [14]) and a policy network implemented by an artificial neural network.

Section 3 contains the main discussion of how a policy network could be a useful representation for navigation (§3(b)) and 3D scene structure (§3(c). Before that, §2 describes the difficulty of finding a neural mechanism for transforming retinotopic signals (wholesale) into an ego- or allocentric reference frame (and raises the question of whether this is a desirable thing to do). Finally, in §4, I discuss some of the questions that might arise from the policy network proposal.

## 2. Three-dimensional coordinate transformations in a moving observer

The main purpose of this paper is to consider how a policy network could be used to represent a 3D scene. First, it is worth reviewing the alternative. It is commonly suggested that the cortex is able to carry out a chain of coordinate transformations from retinal coordinates (e.g. in V1) to a variety of ego-centric coordinate frames, particularly in posterior parietal cortex [16] and then to a world-based coordinate frame in the hippocampus and surrounding cortex [17,18]. In this section, we briefly consider some of the difficulties associated with this proposal before considering an alternative in §3(a). Figure 1*b* illustrates one of the many complexities. Firing rates of neurons in retinotopic coordinates must be copied or transferred in some way to neurons in another cortical area where the receptive fields are stable in head-centred coordinates. Then, firing rates from that area must be copied or transferred in some way to a different brain region where receptive fields are stable in world-centred coordinates. Byrne *et al.* [19] show in detail how complex this process must be, with an input representation duplicated many times (20 in their example) so that one can be chosen on the basis of a head-direction cell input. Then these firing rates, and only these, are transferred to the output representation. But the problem becomes much more complicated when the animal not only rotates their head or eyes but also moves (translates). Figure 1*b* shows this. There must be a different rule for transferring firing rates for objects at different depths because the relationship between retinotopic coordinates and egocentric coordinates as the observer moves depends on object distance. It is not sufficient simply to have a separate mapping for each retinotopic location and each preferred disparity of V1 neurons: there also needs to be a different mapping for each direction of movement the observer could make. While there is evidence that this updating is achieved in the absence of visual feedback [20], at least to some extent [21], there are no detailed proposals for transferring firing rates wholesale to an area with a different coordinate frame as illustrated in figure 1*b* and as described for the case of head rotation by Byrne *et al.* [19].

One possible simplification is to update only a few objects (discussed by [20,22–24]) rather than carrying out a wholesale transformation of all the firing rates that describe the visual scene in retinotopic coordinates. There are neural network implementations to show how a single object could be encoded in head-centred coordinates for different eye positions (e.g. [25]) but, in essence, this involves the same duplication of firing rates that Byrne *et al.* [19] proposed (see fig. 2 in [25]) and there are no equivalent proposals that deal with observer translation. Another simplification of the problem is to only update a single vector, such as a reach vector, in response to a head movement and there are neural network models that achieve this [26].

It has been pointed out that transforming *all* the spatial information about the scene from one coordinate frame to another may not be the goal that the brain is trying to achieve. Pouget *et al.* [25] say that: ‘The question now arises about whether the notion of a Euclidean frame of reference ·· · is the best way to characterise these neural representations’. Even in the 1980s, Zipser & Andersen [27] commented ‘But it is also possible that the final spatial output could only exist in the behaviour of the animal’. In summary, photogrammetry (the process of computing a reconstruction of the scene from images) is highly complex [28], and it is hardly surprising that there are, currently, no suggested neural implementations to recreate it.

Despite the emphasis in the literature on putative visual coordinate transformations in the parietal cortex, it is important to bear in mind that the primary neurophysiological evidence for a world-centred representation (place and grid cells [17,18]) does not support allocentric representation of the *visible* scene since, by definition, the current location is not visible. Figure 1*c* illustrates the two outputs from photogrammetry, i.e. the process of recovering the 3D structure of the scene and the location and orientation of the camera given only a set of images that the camera takes as it moves through the scene [29]. Place cell, head-orientation and grid cell responses carry information about the location and orientation of the camera but they do not deliver a 3D representation of the *scene* as photogrammetry does. Thinking about the operations in parietal cortex and the hippocampus as something analogous to the computation involved in photogrammetry may be the wrong approach. An alternative is outlined in the following section.

## 3. Policy networks for 3D vision

### (a) A policy network for pure rotation of the eye or camera

The simplest policy network to consider for vision is one for pure rotation of the eye or camera. Camera rotation provides no information about the 3D structure of the scene but, nevertheless, I will argue that it forms a fundamental basis for describing scene structure and for navigation. Babies spend many weeks looking around a scene with no opportunity to move to a new location of their own accord, but they can move (i.e. rotate) their eyes. They learn to relate the images they receive before and after a saccade. If there are five objects that the baby might want to look at then there are 5×4=20 saccades between them (in general, n(n−1) saccades for *n* points). This can be described as a policy network, *π*(**a**|**s**, **g**), where **a** is a set of actions (in this example, the set of 20 saccades), **s** is a set of sensory states (in this case the five retinal images of the five objects the baby fixates) and **g** is a set of goals (signalling the desire to look at one of the other four objects). The form of this vector, **g**, does not have to be the same as the retinal images, **s**, it just has to ensure that the same action does not occur every time the baby looks at a particular object, i.e. (**s_t_**, **g_t_**) is a richer signal than (**s_t_**). For example, if one tried to set up a system that performed a complex sequence of actions, like making a cup of tea [30], where each action was triggered by the retinal stimulus, this would run into trouble if, at two stages in the activity, the same retinal stimulus occurred but a different action was required (e.g. before and after the kettle boiled). A minimal vector, even a scalar, *g*_*t*_, could be sufficient to distinguish these two situations and hence lead to different outputs (e.g. ‘look for teapot’ or ‘pick up the kettle’).

In the case of eye movements, this set of stimulus+goal contingencies (or policy network) together form a representation of the relative visual direction of the *n* objects: i.e. this is a type of egocentric representation of visual direction [31,32]. It differs from more traditional ways of thinking about egocentric representations [33], and it leads to an interesting way to consider the relationship between egocentric and world-based representations. We will see in §3(b) and 3(c) that this representation of visual direction (in which the camera/eye only rotates) is the most appropriate starting point for considering (a) a representation of the location of objects and (b) a representation of the location of the observer. This may seem odd, given that pure rotation of the camera about its optic centre provides no information about the 3D structure of the scene but in both cases it provides a world-based foundation for the representation.

### (b) A hierarchical address for the location of the camera/eye

Having considered a policy network for pure rotations of the camera/eye, we can now look at the changes that occur when the eye translates. These provide information about both the spatial structure of the scene and how the camera/eye is moving relative to the scene.

**Figure 2.**
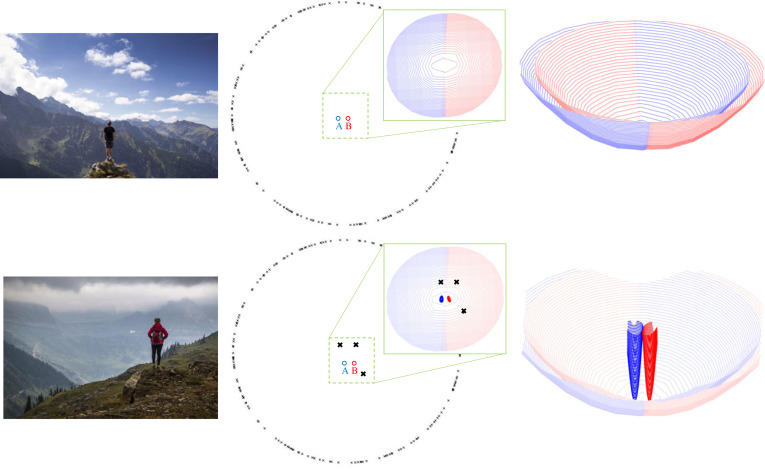
A hierarchical system for defining location. In the upper photo, all the visible objects (mountains) are very distant. This means that any visual information will change very slowly as the observer moves (translates). In [34], this visual information is a vector that lists the angle between all possible pairs of objects, *ε*. The difference between this signal measured at location *A* (i.e. *ε*_*A*_), and at other locations changes very gradually (shown in blue) and so it is hard to distinguish location *A* from location *B* using visual information (the red surface shows the difference between *ε*_*B*_ and *ε* measured at other locations). These functions become very much more spatially restricted when three closer objects are introduced into the scene (black crosses), as shown in the bottom row. Coarse and fine estimates of location are shown together for comparison. For details, see Muryy *et al.* [34]. (Images of mountains are licensed under Creative Commons CC0 - Public Domain). (Online version in colour.)

Figure 2 shows a hiker looking out at mountains with nothing in their view that is close to them. Visually, this situation is equivalent to the pure rotation of the camera in §3(a): even though the hiker *can* move (translate), this makes no difference to the retinal images because the mountains are so distant. Only rotations of the camera/eye affect the image.

Another way to describe this is that the policy network for saccades between distant points in the scene remains valid over a wide range of locations. The region of space over which this set of saccades is (approximately) correct, covers a large area. This is shown in the upper row of figure 2 which shows, for a set of distant scene points, how slowly the set of angles at the camera/eye changes with changes in location of the camera/eye. These plots are made by listing all the angles between *n* points in a 2D scene as a $R^{n(n-1)}$vector, *ε*, and plotting the Euclidean distance between that and the reference vector, *ε*_*A*_, measured when the camera/eye is at point *A*.

The lower panel in figure 2 shows what happens when three near points are added into the scene. The angles between these and other points now change much more rapidly as the camera/eye translates (i.e. as the person moves or views the scene with two eyes); in other words, there is now a significant parallax signal. As a consequence, it is much easier to tell whether the observer is at *A* or *B* (i.e. the blue and red surfaces showing the negative log likelihood of being at *A* or *B* are now much steeper than they were in the upper panel of figure 2). Taken together, these examples demonstrate the principle of a hierarchical method to provide the address of a location. Instead of using a Cartesian coordinate system, with an origin, fixed axes and no hierarchy, the ‘address’ of *A* is the same as the ‘address’ of *B* at the coarsest level but differs at the finest level (just like the postal address of two houses in the same street). The policy network comprising the saccades between distant features defines this coarse scale address and, as nearer features are added, the information about the location of the camera/eye becomes more tightly defined.

This system for defining location is also compositional in the sense that a crude estimate of location can be refined. In many places, observers do not need a very fine gradation of location about where they are (i.e. a large number of slightly different hypotheses). Instead, a rough one will do, e.g. ‘I am in the middle of the garden’. In other situations, when slightly different locations need to be distinguished for the task, it is easy to add new hypotheses and hence ‘split’ categories. This process of adding location hypotheses can be extended to both finer and coarser scales, almost without limit, widening the spatial range of the representation or providing finer granularity where required by the task.

Currently, this type of hierarchical organization is not present in spatial representations that are developed through reinforcement learning. For example, Muryy *et al.* [34] have analysed the representation built up by an agent that learns to navigate from a current image to a goal image [35]. The representation contains very little information about the agent’s location. Future adaptations of reinforcement learning for navigation could be improved by incorporating a hierarchical, compositional scheme of the kind described above (figure 2).

### (c) Structure of the world: a reference frame for parallax

In computer vision (photogrammetry, e.g. figure 1*c*), reconstructing the scene and recovering the location and pose of the camera are complementary components of the same calculation. We considered recovery of camera/eye location in the previous section. In this section, we concentrate on the description of scene structure. Just as we saw for observer location, the idea of using a coarse-to-fine set of hypotheses removes the need for a coordinate frame.

**Figure 3.**
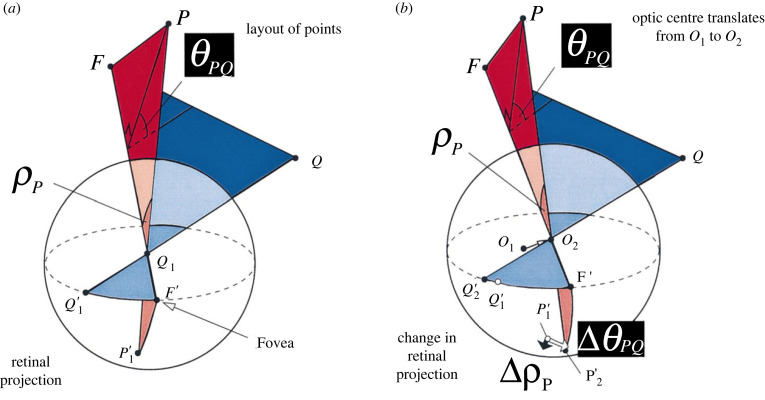
Visual direction and optic flow. (*a*) Retinal location provides information about the visual angle of points relative to the fixated point which are readily described in a polar coordinate frame (retinal eccentricity, *ρ*_*P*_, and meridional angle, *θ*_*PQ*_). If a camera/eye only rotates about its optic centre, these polar angles are the only sensible way to describe the relative visual direction of objects in the scene, because they form a coordinate frame that is independent of the direction the eye is looking (i.e. independent of the fixated point). (*b*) When the camera/eye translates (or, equivalently for binocular vision, going from the left to the right eye) there are changes in these angles (change retinal eccentricity, Δ*ρ*_*P*_, and change in meridional angle, Δ*θ*_*PQ*_). Thus, the 2D coordinates of optic flow and binocular disparity are derived from the natural coordinate system (perhaps the *only* logical coordinate system) used for defining visual direction in a freely rotating camera/eye. (Reproduced from [36] with permission from Elsevier.). (Online version in colour.)

At the coarsest scale, the saccades between distant points in the scene (saccades that do not change despite observer head movements) not only define the crudest ‘address’ of the observer (§3(b) and [34]), but also carry information about the location of these points: points joined by these unchanging saccades must be distant (figure 3 and [36]). This policy network, the one relating all the distant points in the scene, is unaffected by rotation of the camera/eye and (to a first approximation) unaffected by translations of the camera/eye. In this sense, it is world-based (i.e. independent of camera/eye movements). One might, therefore, call it an allocentric representation. It is, of course, also an ego-centric representation as it refers to angles subtended at the optic centre of the camera/eye. This sounds contradictory, but only if one is trying to think about a Cartesian frame of reference. The idea introduced in §3(b) of a distribution in space that corresponds to the estimated location of the optic centre is helpful: when that region expands to a very large range (as happens if the observer is viewing distant points figure 2), then this type of representation is, in practice, both ego- and allocentric.

Nearer objects change their visual direction relative to the set of distant objects as the observer moves. We saw how this provides a more precise estimate of the observer’s location (figure 2). The parallax of near objects relative to distant points also signals that they are close without necessarily using a Cartesian coordinate frame [37]. A baby might try to reach out and touch objects in this category, before they had learned a more sophisticated representation of space. In this sense, learning about the sensory contexts that correspond to near and far distances can be gradually refined, in a compositional manner, just as we discussed in §3(b) for refining a description of the observer’s location.

Marr’s 2$\frac{1}{2}$-D sketch [38,39] assumed that observers had an approximate representation of the layout of a scene, independent of the observer’s eye movements. Although Marr only described this for a restricted field of view, the idea can be applied to an egocentric representation of the scene all around the observer including information about the visual direction of objects, their relative distance, the slant of surfaces and the relief of points on a surface. ‘Information about’ does not necessarily imply internal consistency between all aspects of the stored information unlike a Cartesian representation of the scene, where consistency is a defining characteristic. Veridicality is a quite separate issue: a Cartesian representation may be a distorted model of the world but it must be consistent across tasks to count as Cartesian [4,21,40,41]. Consider, for example, a person who views a scene binocularly and moves a few centimetres in different directions, enough to gain useful parallax but not far enough to change the visual directions of objects substantially. The reference frame for visual direction is a relative one (the saccades linking pairs of points) and the precision for programming these saccades and judging relative visual direction corresponds to, say, a Weber fraction of 3–5%. The point is that information (in this case relative visual direction) is stored in a way that is appropriate for the action that might depend on it (in this case a saccade) but without the necessity for consistency checks.

Distinguishing near from far objects using simple heuristics, as described above, does not require a coordinate frame. Equally, many judgements about surface slant can be made without using either an ego-centric or a world-based coordinate frame. The simplest slant to define (and hence to be the origin of the slant metric) is one where the surface is orthogonal to the line of sight (because expansion or contraction of the image is the only possible local flow, at least for small translations of the camera/eye). Other slants can be measured relative to this baseline [42,43].

Finally, to measure the depth relief of points, the sensible reference frame is the surface on which these points lie rather than, as is often assumed, the ‘fixation plane’ (a plane through the fixation point that is parallel to the inter-ocular axis). It is sensible because, unlike the fixation plane, the local surface provides a world-based reference frame against which to measure depth relief and, unlike the fixation plane, this is independent of observer head and eye movements. There is psychophysical evidence that this reference frame determines (i) judgements of stereo correspondence, (ii) perceived depth and (iii) the plane with maximum sensitivity to depth changes [44–48]. So, the depth of points can be measured relative to a local surface; local surface slant can be measured relative to a plane that is orthogonal to the line of sight; the line of sight (i.e. the visual direction of a surface) can be measured relative to other points in the optic array; and optic arrays themselves can be organized in a hierarchical manner, as described in §3(b). This hierarchical set of relationships takes us from local relative depth through egocentric spatial layout to allocentric representation without any resort to 3D coordinate frames [36,49]. Although this description may sound radical, it need not be so different in practice from a reconstruction-based representation. In fact, the psychophysical predictions of the two hypotheses are often very similar, so carefully designed experiments are required to tease the two apart [4,21,41,50].

One issue that we have not considered is how a policy network for 3D vision might be learned. Most learning by animals involves a compositional representation, i.e. we learn a response to a broad category of stimuli and then, as we learn more, we divide that category up, refine our representation and respond in different ways to different sub-categories. Section 3(b) gave an example of this hierarchical learning of location that can be tailored to task demands. This is quite different from a Cartesian system, where the resolution and coordinate frame are predetermined and are not necessarily related to learning.

In summary, we have considered in this paper the idea that a policy network describes the way that a set of stored actions can move a state vector across a manifold of potential states. In §3(b), we saw how the current state could be described using a hierarchical address on that manifold. Now, in §3(c), we have re-described knowledge about the 3D structure of the scene as knowledge about the image that will be received as the camera/eye moves through the world or, in other words, storing a policy network and knowledge about the location on the manifold to which that action moves the current sensory state. Instead of a reconstruction of the scene and camera/eye location, we store information about the manifold of potential states and the rules (or policy network) for traversing it.

Binocular vision, which we have rarely mentioned so far, provides a prediction of where the current state would move to on the manifold if the left eye moved in space to the location of the right eye. We have been considering the general case of a state, **x**_**t**_, moving across a manifold of states, but binocular vision in a static observer amounts to only two points on that manifold. As such, binocular vision is probably best understood once the properties of the manifold are well established.

### (d) Disproving a policy network

An attraction of the policy network idea as a hypothesis for 3D vision (or other aspects of perception) is that it is remarkably difficult to disprove (which is presumably preferable to being *easy* to disprove). But it is not impossible. Every computing/Turing machine produces an output on the basis of a current state but the proposal here is that that state does not need to be an elaborated one as, for example, when photogrammetry generates a set of 3D coordinates before a decision is made. Instead, the sensory data can be left in a much more raw form and used in different ways according to the task at hand. Disproof would come from showing that that is not the case. We will see one example below in relation to grid cells [51] where a rigorous demonstration of grid-like consistency across two rooms would disprove (or at least be highly problematic for) the policy network hypothesis. The same goes for the neurophysiological mechanisms proposed by Byrne *et al.* [19] (§2) for wholesale 3D coordinate transformations between ego- and allocentric reference frames. If that were really shown to occur in the brain, it would be wholly counter to the policy network idea.

## 4. Discussion

The ideas set out here raise a variety of questions. I discuss two of these below (see also [7]). One is the claim that grid cells [18] are known to provide a Cartesian map and so there is no need to propose a non-Cartesian alternative. Another is that there are tasks that *must* require a 3D reconstruction and could not be done using only a set of reflexes (a policy network). The first is readily dismissed, at least in its crudest form. The second less so, because it is always possible to think up more and more complex tasks, but I discuss one example of the way in which latent representations can take the place of a 3D reconstruction in explaining human performance.

### (a) Grid cells perform this function already

It is often said that grid cells provide a map [51,52] to tell the animal where it is whereas, in many ways, the opposite is true. A regular grid-like pattern of firing does appear similar to the grid on an *Ordnance Survey* map, or to lines of latitude and longitude, but the point about gridlines on a map is that they are uniquely labelled and can be used to define a location. By contrast, the firing of a grid cell is entirely ambiguous so it cannot be used on its own (or even in combination with the other two grid cells at this scale (and, at a given scale, there are effectively only three grid cells)) to determine the animal’s location in the way that a grid reference does on a map. This problem is discussed and potential solutions proposed [52] but, despite this, there remains a strong tendency for authors to assume that the regular pattern of firing of grid cells is indicative of a map with a grid-like coordinate system.

There have been simulations showing that grid-like pattern of spatial responses can emerge in networks, e.g. using as input the translational and rotational velocity of the agent (proprioceptive signals). Banino *et al.* [12] showed this and demonstrated that a trained network of this sort could be used to achieve a variety of navigational tasks using the ‘grid cell’ signal. But the current state vector used in this training was a high-dimensional vector ($R^{512}$) with only 129 of these elements showing grid-like behaviour and their periodicity is, if anything, a hindrance rather than a help in defining location unambiguously. The authors describe what has been learned as a policy network; it is not a Cartesian map.

Another example helps to highlight the difference between policy networks and a Cartesian reconstruction. Carpenter *et al.* [51] describe an experiment in which two rooms are connected by a corridor and, initially, the pattern of grid cell firing in both rooms is very similar. As the rat becomes more familiar with the second room, the grid cell firing there changes and this change ‘could reflect’ a globally consistent grid pattern covering the two rooms, the authors say. A strong version of this claim is that if the orientation and length of the corridor were suitably arranged, the grid cell firing should *return to being the same* in the two rooms again for some suitable corridor length (or, as a milder prediction, there should be some periodicity in the cross-correlation with the grid-cell firing in the original room as the corridor length is varied). This would be truly remarkable evidence and would be strongly counter to the claim made in this paper that the sensory signals to which the grid cells respond should just be considered as one among a long list of sensory inputs (**s**) that help to define a location. It would show, instead, that some kind of ‘top down’ imposition of a regular structure (like latitude and longitude) was influencing the pattern of grid cell firing. This is what is implied by the notion of a globally consistent grid cell firing pattern and, if it were shown to be correct, it would disprove the type of argument advanced here based on policy networks.

### (b) ‘I can think of a problem you can’t solve’

Another possible objection is that it is possible to think of an activity, such as moving a piano through a doorway, where it is (at least in the mind of the questioner) hard to imagine that anything so simple as a policy network could solve it. It is only fair, when considering an issue like this, to examine two proposed solutions side-by-side, where each is specified from the algorithmic level down to the proposed neural level. The latter is important because, as we have discussed in §2, the neural implementation of Cartesian-based solutions is currently unclear (and perhaps its Achilles’ heel) while that for a policy network is potentially straightforward. In our virtual reality laboratory, we investigated a task that has some similarities to the piano-mover’s question in that it requires a mental manipulation of a 3D structure in order to solve it. Participants viewed a scene, then walked through a corridor to a location where they had to point (without visual feedback) to several objects that they had originally viewed [21]. We found that although participants could point in roughly the correct direction, they showed large, systematic biases in this spatial updating task and their responses were difficult to explain on the basis of a distorted internal model of the scene. We repeated this finding in the real world. Many have assumed that a 3D model is required in order to do this type of spatial updating task, but that is not the case. Recent findings in reinforcement learning [53] have shown that it is possible for an agent to generate an image (and hence point to an object) from a novel location in a novel room if the agent is given only one or two ‘seed’ images of the room from a quite different vantage point (as was true for the participant in our experiment). This demonstration is important because it shows how tasks that seem as if they must require the brain to use a 3D representation, in fact do not. Building a latent representation through many previous experiences, and being able to generalize in appropriate ways, allows remarkable performance without involving any 3D geometry.

Clearly, the examples I have considered in this paper are very simple (such as making a saccade from one object to another or moving the head), whereas humans engage in tasks that have many levels of complexity, including navigational tasks. One possible area of speculation is about how motivational input (here, **g**) could contribute to the hierarchical organization of complex tasks, including ‘chunking’ [54], but that is beyond the scope of this paper.

## 5. Conclusion

At the broadest level, there are two types of idea about the representation that might underlie 3D vision. According to one, all the complexity of the problem is concentrated on the neural mechanics of building an internally consistent replica of the world that correlates with perception and then using this reconstruction to generate a motor response. An advantage of this approach is that it is easy to imagine, at least at a superficial level. However, as we have explored in §2, there are severe problems when it comes to specifying the details of a neural mechanism. The argument is turned on its head when it comes to a policy network which is, in essence, a long list of reflexes. There is little doubt that this is something the brain can store. A disadvantage is that it is much more difficult to imagine how this could be the basis of our perception of a 3D world (or, indeed, smells, sounds, colour, anguish). But, neuroscientists can be prone to Philosophers’ Syndrome: ‘mistaking a failure of the imagination for an insight into necessity’ [55]. The speculations in this paper will, I hope, stimulate this process of imagination. They point out how a whole range of judgements that we tend to think of as involving scene reconstruction can, instead, be described in terms of a changing image: the visual direction of objects, their depth, the slant and relief of surfaces, the spatial layout of a visible scene (§3(c)) and, beyond that, navigation (§3(b)). The argument advanced here is that it is worth that effort of imagination because, if the ideas hold up to scrutiny, we would be closer than we might have thought to a neurally plausible account of 3D vision.

## Data Availability

This article has no additional data.
